# Acoustic and linguistic influences on rise-time modulations in natural English speech: evidence from a sensorimotor synchronization paradigm

**DOI:** 10.3389/fpsyg.2025.1544948

**Published:** 2025-07-18

**Authors:** Chia-Yuan Lin, Tamara Rathcke

**Affiliations:** ^1^Centre for Cognition and Neuroscience, Department of Social and Psychological Sciences, University of Huddersfield, Huddersfield, United Kingdom; ^2^Department of Linguistics, University of Konstanz, Konstanz, Germany

**Keywords:** amplitude envelope, rise-time, sensorimotor synchronization, P-centre, maxD, speech rhythm

## Abstract

Modulations of amplitude rise-time are considered fundamental to speech rhythm. However, rise-time is a holistic measure of the waveform shape and, as such, may be influenced by a variety of factors, potentially obscuring relationships between speech rhythm, signal acoustics, and linguistic structure. To address the factors that can modulate the rise-time of amplitude envelopes in English and the impact that rise-time modulations may have on rhythm perception in natural connected speech, we recorded 52 English sentences produced by a native female speaker and examined the effect of metrical weight, nucleus duration, average intensity, syllable onset complexity and sonority on rise-time duration in these sentences. As expected, amplitude rise-time was reflective of both acoustic-phonetic (nucleus duration and average intensity) and linguistic (onset complexity and metrical weight) factors. In addition, we conducted a sensorimotor synchronization experiment in which 31 native English speakers tapped in time with the beat of the recorded sentences. Analyses of synchronization showed that rise-time played a limited role in explaining rhythmic variability in these data. Taken together, the present findings indicate that rise-time cannot be straightforwardly mapped onto a specific linguistic function or a specific feature of the acoustic speech signal and is, therefore, difficult to interpret meaningfully. These results highlight a complex relationship between rise-time and speech rhythm and raise critical implications for speech rhythm research based on holistic acoustic measures such as rise-time.

## 1 Introduction

Modulations of the amplitude envelope, defined as ‘fluctuations in the overall amplitude at rates between 2 and 50 Hz’ (Rosen, 1992, p. 368), are inherent to the acoustics of speech signals (Myers et al., 2019). Previous research has suggested that such amplitude modulations carry rhythmic information with periodicities arising at different time scales (Cummins and Port, 1998; Leong and Goswami, 2014; O’Dell and Nieminen, 1999; Tilsen and Arvaniti, 2013; Tilsen and Johnson, 2008). Periodicities on the temporal scale of 3–5 Hz (or 200–300 ms) appear to prevail in all speech signals, irrespective of the language of the recording, and are considered among the clearest rhythmic cues representative of the syllable rate in speech (Leong and Goswami, 2014) while faster periodicities (up to 50 Hz, i.e., occurring at the rate of 20 ms) are more language-specific and are thought to correspond to phonemic information (Leong and Goswami, 2014).

Amplitude envelopes are considered critical for speech rhythm perception (Cummins and Port, 1998; Leong and Goswami, 2014; O’Dell and Nieminen, 1999; Tilsen and Arvaniti, 2013; Tilsen and Johnson, 2008), though empirical investigations of amplitude modulations in natural speech remain relatively rare (cf. Braun, 2026). Individual sensitivity to the duration of signal rise-time in short non-linguistic stimuli is known to correlate with – and even predict – first language acquisition and development. For example, dyslexic adults (Hämäläinen et al., 2005; Leong et al., 2011; Thomson et al., 2006; Van Hirtum et al., 2019) and children (Goswami et al., 2010; Huss et al., 2011; Richards and Goswami, 2019) differ from typically developing listeners of the same age in their perception of signal rise-time in tones or one-octave noise bands. More specifically, dyslexic listeners, unlike age-matched controls, have difficulty distinguishing short sounds that differ in their rise-times and tend to perceive them as if they had identical rise-times.

Longitudinal studies have indicated that an individual sensitivity to modulations of rise-time in pure tones during infancy predicts vocabulary knowledge later in childhood (Kalashnikova et al., 2019). Moreover, the perception of amplitude rise-time in short monosyllables like [ba] and [wa] appears to take a long time to develop and is still not adult-like in 4- to 5-year-old children (Carpenter and Shahin, 2013). Research using algorithms to enhance local envelope information in natural spoken sentences has shown positive effects on auditory processing skills in children with a family risk of developmental dyslexia (Van Herck et al., 2022). Such rise-time enhancements have been suggested to modulate neural responses in the delta band, which are related to speech rhythm processing (Lizarazu et al., 2021). Taken together, these findings indicate that rise-time may play a critical role in speech rhythm perception.

**Figure 1:**
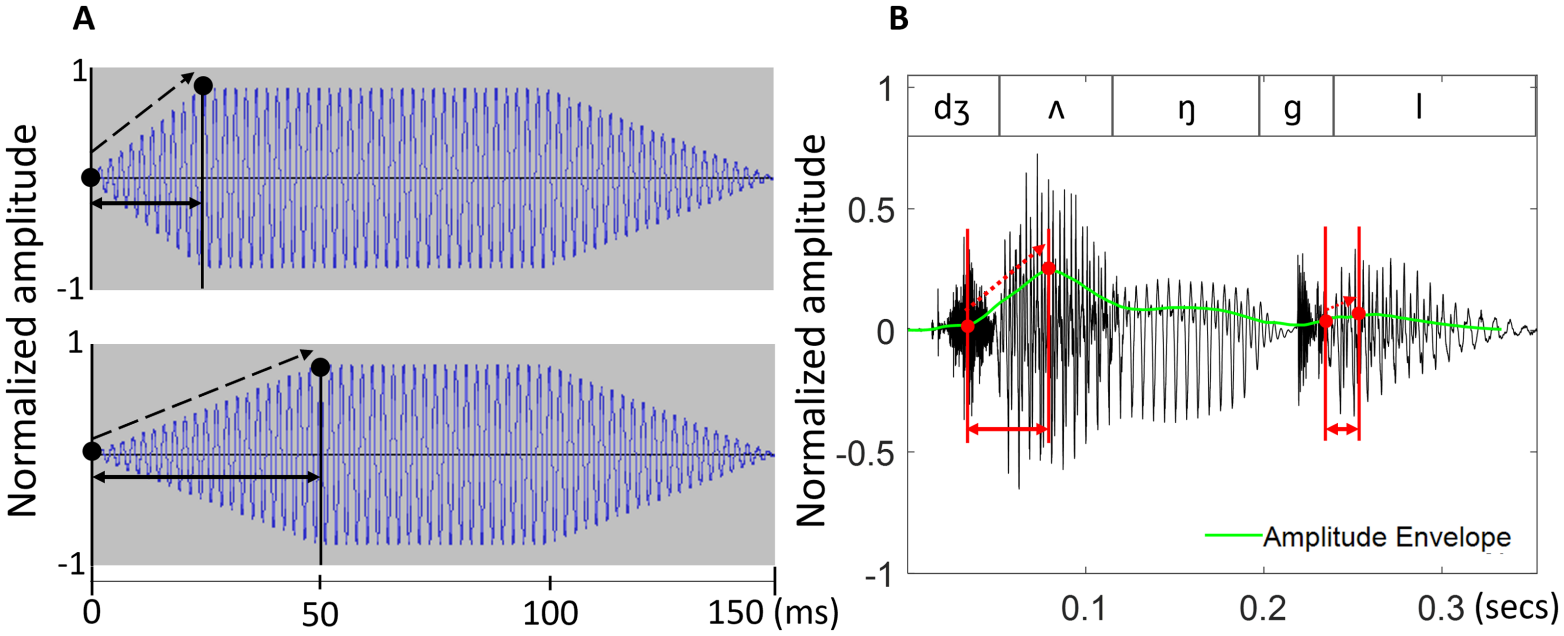
An example of rise-time in **(A)** two tonal stimuli and **(B)** the bysyllabic word ‘jungle’.

In these studies, rise-time usually refers to the duration of the local changes in the amplitude envelope and measures the interval between the local minimum and maximum of the energy contour at the onset of a sound (Greenberg, 2005, see Figure 1). Amplitude rise-time has frequently been discussed as the key to understanding speech rhythm for three main reasons. First, even though rhythm in speech is usually subtler than it is in music (Dalla Bella et al., 2013), it becomes more apparent with regular energy changes forming a rhythmic pattern, as, for example, in nursery rhymes (Leong and Goswami, 2014). Such energy changes are thought to cue primarily syllable-level variation in the acoustic speech signal, and previous research has suggested that individual variability in rise-time perception reflects the ability to clearly perceive lexical stress placement and successfully differentiate between stressed and unstressed syllables (Leong et al., 2011). As the example of the word ‘jungle’ in Figure 1B shows, the rise-time of the first, stressed syllable is notably longer than that of the second, unstressed syllable. However, other accounts suggest a rather minor role of the amplitude envelope as a cue for the perception of lexical stress (Rosen, 1992), especially given that acoustic cueing of lexical stress demonstrates large cross-linguistic differences and can be expressed primarily by spectral rather than temporal cues (Gordon and Roettger, 2017; Ladd and Arvaniti, 2023).

Second, it has been proposed that properties of amplitude rise-time offer an acoustic scaffold for speech perception, guiding the alignment of the brain’s internal neural oscillations with the external amplitude modulation patterns of spoken language (Goswami, 2019; Greenberg, 2005; Leong et al., 2014; Leong and Goswami, 2014). In support of this view, some studies have provided empirical evidence that dyslexic adults exhibit atypical neural entrainment at syllabic rates (assumed to lie at timescales of 2.5–12 Hz), but not at phonemic (12–40 Hz) or linguistic stress (0.9–2.5 Hz) rates (Leong and Goswami, 2014).

Finally, properties of amplitude rise-time have also been central to the discussions of the so-called ‘perceptual centres’ (P-centres), or subjectively experienced syllable onsets that do not necessarily align with their acoustic onsets (Marcus, 1981; Morton et al., 1976). It is the P-centre – and not the syllable onset – that has been suggested to govern the perception of speech rhythm (Marcus, 1981; Morton et al., 1976). The P-centre of a given syllable is sometimes said to fall somewhere between its acoustic onset and the amplitude maximum located in the syllable nucleus, typically a vowel (Leong and Goswami, 2014; see Figure 1B for two examples of syllable nuclei), with envelope properties around the nucleus onset being particularly relevant for determining the exact P-centre location (Marcus, 1981; Morton et al., 1976; Scott, 1998) and onset rise-time or onset consonant duration playing a particularly important role (Marcus, 1981; Scott, 1998; Šturm and Volín, 2016). In phonology, syllables are viewed as being built around sonority maxima located in the nucleus (Clements, 1990) and acoustically expressed by a local energy maximum (Morgan and Fosler-Lussier, 1998; Wang and Narayanan, 2007), with only a few, typologically rare exceptions (Ridouane, 2008), though low-sonority nuclei are also possible in connected speech (Fujimoto, 2015; Nolan and Kerswill, 1990).

In (A), two pure tones differ only in their rise-time (indicated by solid black arrows) and rise-slope (indicated by dashed arrows). The example in the upper panel is a tone with a 25 ms rise-time and a steeper rise-slope while the example in the lower panel is a tone with a 50 ms rise-time and a shallower rise-slope. In (B), the rise-time of both syllables in ‘*jungle’* is shown in red, stretching from the start of the syllable to the maximum point of the amplitude envelope. The amplitude envelope, shown in green, is calculated as a smoothed energy contour from the original sound wave (see Methods for more detail). The word contains a vocalic (/ʌ/) and a consonantal (/l/) nucleus. Note that rise-time and rise-slope are orthogonal – that is, a longer rise-time does not necessarily imply a shallower rise-slope, and vice versa.

Amplitude rise-time has been suggested to encode segment identity rather than lexical stress (Rosen, 1992). For example, shorter rise-time leads to the perception of an affricate rather than a fricative (e.g., /tʃɑ/ rather than /ʃɑ/) (Howell and Rosen, 1983), or a stop rather than a glide (e.g., /bɑ/ rather than /wɑ/) (Walsh and Diehl, 1991). Even though formant transitions predominantly cue consonant identity (Benkí, 2001; Walsh and Diehl, 1991), evidence exists for consonant identification in the absence of these transitions, based solely on the properties of amplitude rise-time (Remez et al., 1981). In line with this research, dyslexic children perform well on consonant discrimination (e.g., between /bɑ/ and /wɑ/) despite shortened formant transitions, but are significantly impaired when rise-times vary (Goswami et al., 2011).

So far, research on the role of rise-time in speech perception has mostly focused on short words or artificially created speech sounds (but see recent studies on ‘speech edges’ in amplitude envelopes, Lizarazu et al., 2021; Van Hirtum et al., 2019). Little is known about the acoustic and linguistic factors that may modulate rise-time variability in natural connected speech (cf. Braun, 2026). Given the complexity of linguistic phenomena potentially encoded in amplitude rise-time (Rosen, 1992; Scott, 1998), it is imperative to pinpoint those aspects of rise-time that have high relevance for rhythm perception in natural speech. Our previous study examined a small set of naturally spoken sentences in English (Rathcke et al., 2021b). We found that there was a small but consistent effect of rise-time on sensorimotor synchronization (SMS) in English participants, tapping along with the perceived beat of sentences. Rise-time influenced the accuracy of synchronization with rhythmically relevant landmarks (as measured by both absolute and signed asynchrony). Specifically, syllables with shorter amplitude rise-times led to smaller absolute asynchronies while syllables with longer rise-times were temporally more anticipated – that is, participants tended to tap ahead of the target. However, the previous study (Rathcke et al., 2021b) was based on only six sentences of English (comprising 42 syllables in total). It therefore remains to be demonstrated whether or not rise-time indeed plays a role in a larger and more diverse set of linguistic materials.

The present study asked (i) what aspects of the linguistic structure and/or acoustic-phonetic signal properties shape the characteristics of amplitude rise-time in natural speech, and (ii) how variable rise-times may influence rhythm perception during sensorimotor synchronization with a linguistically varied set of spoken sentences. The study builds on our previous work into rhythm perception in natural speech by examining sensorimotor synchronization with the beat of looped spoken sentences (Lin and Rathcke, 2020; Rathcke and Lin, 2023; Rathcke et al., 2021b) and extends our previous findings by a closer examination of rise-time.

## 2 Method

### 2.1 Sentence stimuli

Fifty-two English sentences were created so as to vary the type of syllable nucleus and to contrast vocalic and consonantal nuclei, which differ in terms of their sonority and the resulting intensity maxima (Morgan and Fosler-Lussier, 1998; Wang and Narayanan, 2007). In addition, attention was paid to contrasting high-intensity, higher-sonority open vowels with low-intensity, lower-sonority closed vowels in similar consonantal contexts (cf. Bladon and Lindblom, 1981). The sentences also varied in the number and distribution of strong and weak syllables, vowel qualities, and the complexity of syllable structure (see https://osf.io/gdq96/ for the full list of sentences). It was expected that the choice of materials would result in a wide range of rise-times representative of natural connected English speech.

The materials were recorded by a female speaker of Standard British English (in her 30s at the time of the recording). Subsequently, syllable boundaries were manually annotated by a trained phonetician (the corresponding author), following the maximal onset principle (Selkirk, 1981). In total, the materials contained 455 syllables (mean = 8.8 syllables, SD = 1.8 syllables, range: 6 to 13 syllables per sentence).

### 2.2 Acoustic pre-processing

#### 2.2.1 P-centre approximation using maxD

**Figure 2:**
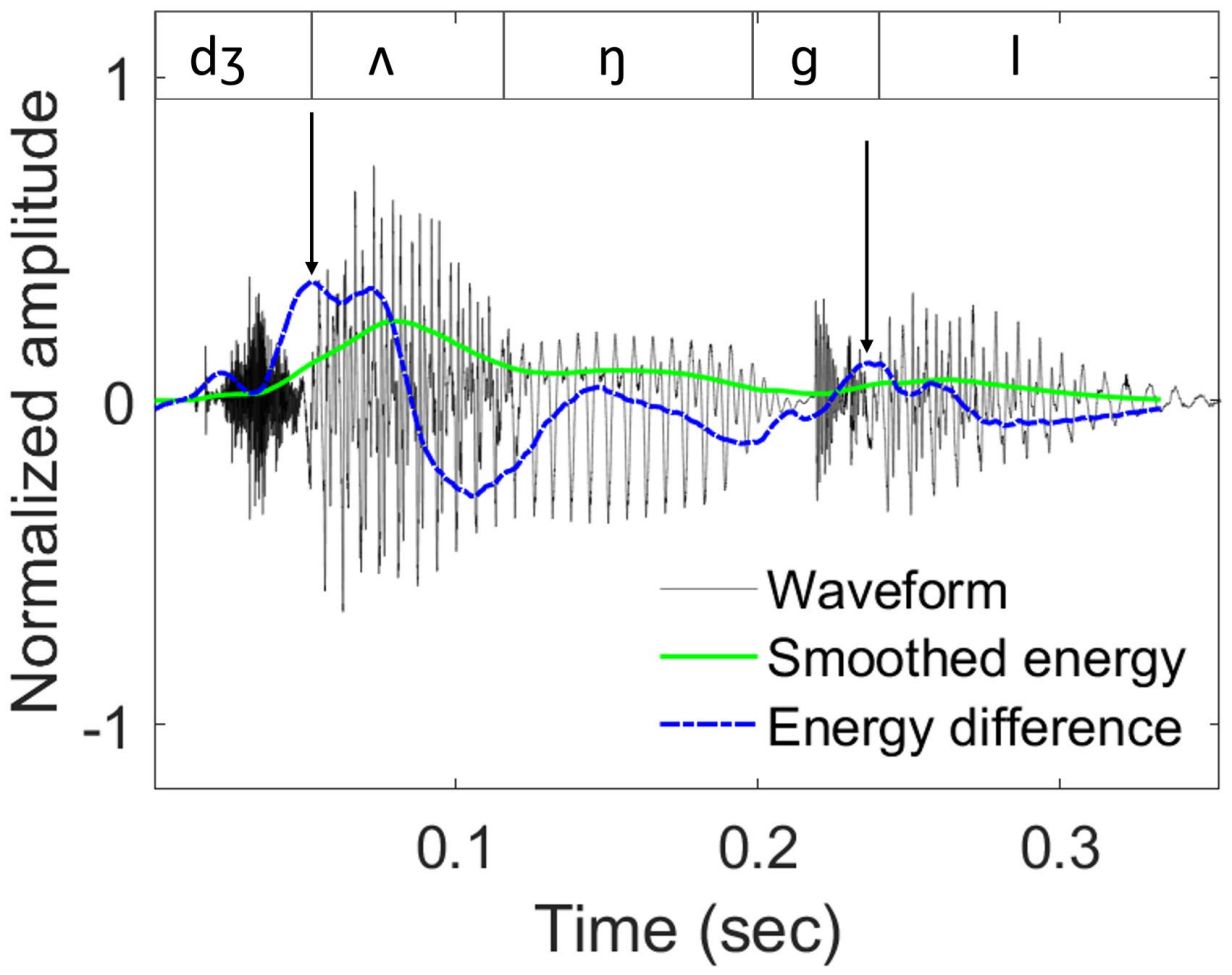
An example of sound waveform, smoothed energy contour, and smoothed energy difference contour.

The closest approximation of the P-centre is currently represented by the location of a maximum energy increase (maxD) within a syllable (Šturm and Volín, 2016). The algorithm to calculate maxD first derives a raw energy contour of a stimulus, by squaring the raw amplitudes and applying a 40-ms window with a 1-ms shift. A smoothed energy contour is then obtained by applying a 6th-order moving average filter and reducing the energy of fricatives (with a zero-crossing rate > 7.5 in 1 ms) to a quarter of the original contour. A similar signal reduction procedure for fricatives was also used by Šturm and Volín (2016). Subsequently, an energy-difference contour is created by calculating energy changes between two adjacent samples of the smoothed energy contour ( $E_{T}-E_{T-1}$ ) and smoothing the resulting energy difference via a 10th-order moving average filter. Finally, maxD is identified as the highest local value in the smoothed energy-difference function for each syllable. The maxD algorithm was applied to all test sentences in the study to calculate the potential synchronization anchors (Rathcke et al., 2021b). An example is shown in Figure 2.

Waveform (dark grey lines), smoothed energy contour (solid green lines), and smoothed energy difference contour (dashed blue lines) for the word ‘*jungle’,* taken from the test sentence ‘*Tom and his uncle explored the jungle*’. Segmental boundaries are shown in the top panel. Locations of the derived maxD landmarks are indicated by black arrows.

#### 2.2.2 Rise-time and rise-slope

**Figure 3:**
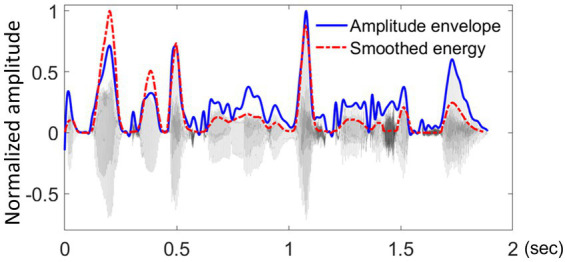
Visual comparison between the smoothed energy contour and the raw amplitude envelope.

For the present study, rise-times and rise-slopes were both derived from the smoothed energy contour. In contrast, our previous work (Rathcke et al., 2021b) derived these measures from raw amplitude envelopes using the Hilbert transform (see Figure 3), which proved error-prone and time-consuming as it required a substantial amount of manual correction. Both procedures delivered similar values for rise-times (*r* = 0.63, *p* < 0.001) and rise-slopes (*r* = 0.53, *p* < 0.001), with the smoothing-based approach having the advantage of requiring only minimal supervision.

Visual comparison between the smoothed energy contour (shown in red, Šturm and Volín, 2016) and the raw amplitude envelope (shown in blue, derived using the Hilbert transform).

The example displays the waveform of the test sentence: *“The broken bottle made a rattle as it fell.”*

Amplitude rise-time was defined as the time between the local minimum and maximum of the smoothed energy contour, spanning the location of maxD. To calculate rise-time, a local energy maximum was first identified for each syllable. The algorithm then searched for a preceding minimum in the locally distributed energy envelope, by comparing the contour slope of adjacent points within a 5-ms interval and identifying a point where the slope dropped by more than 70% compared to the preceding value, which was identified as the local energy minimum. The algorithm generally performed well in locating the maxima and minima in the energy envelope, though some syllables were produced with a relatively flat energy curve, making the exact estimation of the local minimum less reliable and requiring manual correction (4.8% of all rise-times were manually corrected). The rise-slope was measured as the energy difference between the local minima and maxima, divided by their rise-time duration.

#### 2.2.3 Other measurements

To understand the factors that may influence the acoustic properties of rise-time, each syllable was described with respect to a number of linguistic and acoustic-prosodic factors. Properties of syllable onsets included a measure of phonological sonority and a descriptor of onset complexity. Based on the sonority hierarchy (Clements, 1990), a trained phonetician (the corresponding author) assigned each syllable onset a numerical value representing average phonological sonority on a scale from 0 to 9, starting with voiceless stops at 0 and ending with open vowels at 9 (for onset-free syllables, the measure reflected the sonority of the nucleus, see Rathcke et al. (2021a) for a similar approach). Onset complexity was defined as the number of consonants in the syllable onset position, originally ranging from 0 to 3 in the dataset. However, only one syllable contained an onset with three consonants, and this was excluded from further analysis. As a result, the final analyses included onset complexity levels 0 to 2 only. Additionally, two syllables were removed due to issues with intensity measurement, resulting in a total of 452 syllables. Although onset complexity was initially coded as an ordinal predictor, it was treated as a categorical variable in the final analysis to allow clearer comparisons between levels and to avoid assuming a specific trend (e.g., linearity) across levels.

Each syllable was further specified in terms of its metrical weight (strong or weak), the duration of its nucleus, and its serial location within the sentence (ranging from 1 up to 13 in longer sentences). The serial order of syllables in sentences was included to account for prosodic phrasing effects that were found relevant in a previous study (Rathcke et al., 2021b). Intensity was measured in Praat and defined as the mean intensity of a syllable nucleus, divided by the mean intensity of the whole sentence (Boersma and Weenink, 2018).

### 2.3 Participants

Thirty-one native speakers of Southern British English were recruited (20 females; mean age = 20.4 years, *SD* = 2.5 years, range = 18–32 years). All participants self-reported normal reading, hearing, and motor abilities. No personally identifiable information was collected.

### 2.4 Tasks and procedure

Participants were asked to tap along with the beat of the experimental sentences. Five practice trials were given in advance of the formal testing session. The practice sentences were looped with 20 repetitions while the experimental sentences were repeated 15 times (cf. previous studies using finger-tapping with looped sentences, Rathcke et al., 2021b; Rathcke and Lin, 2023). Participants were instructed to tap with the index finger of their dominant hand on a drumming pad (Roland Handsonic HPD-20) placed in front of them. CakeWalk by BandLab was used for both sound playback and tap recording. After the practice session, participants were instructed to start tapping from the third repetition during the formal testing trials. The experiment took approximately 40 min to complete.

All participants gave written informed consent prior to the experiment and were reimbursed for their time and effort. The protocol received ethical approval from the Faculty of Humanities Research Ethics Advisory Group for Human Participants at the University of Kent.

### 2.5 Tapping data pre-processing

**Figure 4:**
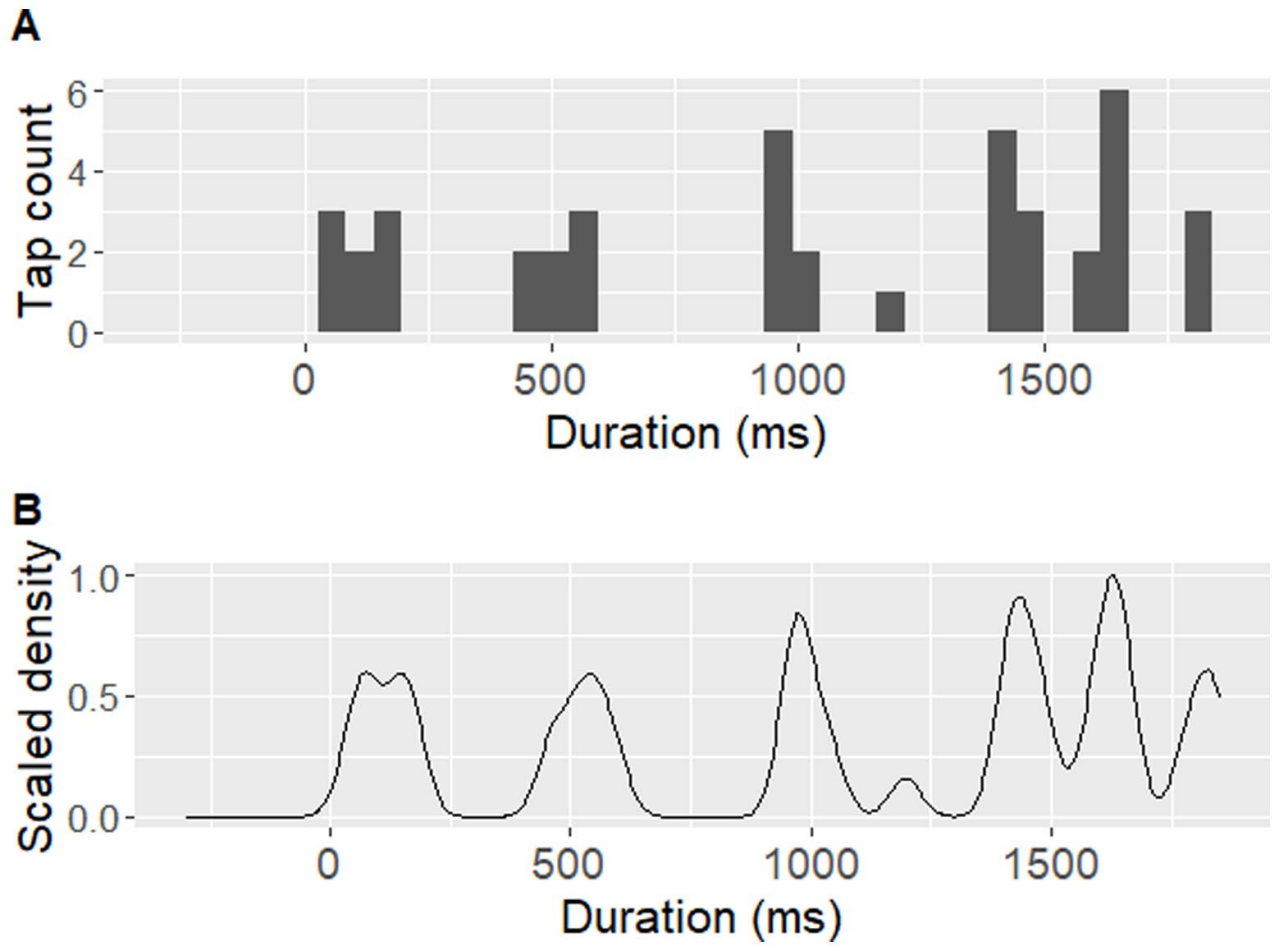
An example of a participant’s tapping performance. An example of one participant’s (P07) tapping performance during the sentence ‘*Tom and his uncle explored the jungle*’. **(A)** displays raw tap counts over time in a histogram with 39 time-unit bins (each corresponding to approximately 46 ms), **(B)** displays Gaussian kernel density estimation for the same tap counts, using a bandwidth of 37.8 ms (corresponding to 1/8 of the default bandwidth based on the *bw.nrd* function in R).

The pre-processing of tapping data focused on identifying those locations within a sentence that frequently led to the individual perception of a beat and consistently attracted a finger tap during repetitions. For this, tapping data were first extracted using the MIDI toolbox in MATLAB (Eerola and Toiviainen, 2004). A 5-ms recording latency from the equipment set-up was subtracted from the raw timepoints of the collected tap locations. All taps collected for each sentence loop were split up into the corresponding repetition cycles. Taps occuring up to 300 ms prior to the onset of a repetition cycle were included. A tapping distribution was then generated for each sentence and participant, by applying a Gaussian kernel density estimation with 1/8 of the default bandwidth from the *bw.nrd* function in R (Scott, 1992; see Figure 4 for an example). Peaks in the tapping distributions were then identified as local density maxima. To be considered a local maximum, a density peak had to reach at least 40% of the largest density maximum identified for the given distribution. A window of 100 ms between adjacent density peaks was employed, to account for physiological limitations in synchronized movement (Repp, 2005). If two tapping peaks were identified within a given 100-ms interval, the higher of the two was chosen.

The described procedure abstracts away much of the variability arising from individual performance on the task and pinpoints the timing of consistently anchored taps, thus highlighting the rhythmically most relevant events. The present approach differs substantially from the analyses typically employed in the study of SMS with simpler and more regular auditory rhythms (Repp, 2005; Repp and Su, 2013) than the non-isochronous rhythms of natural language (Cummins, 2012; Dauer, 1983; Fowler and Tassinary, 1981; Pointon, 1980; Roach, 1982; Uldall, 1971; van Santen and Shih, 2000). The pre-processing of linguistic tapping data and its rationale are explained in more detail in our previous proof-of-concept work (Rathcke et al., 2021b).

### 2.6 Calculation of asynchrony for each syllable

**Figure 5:**
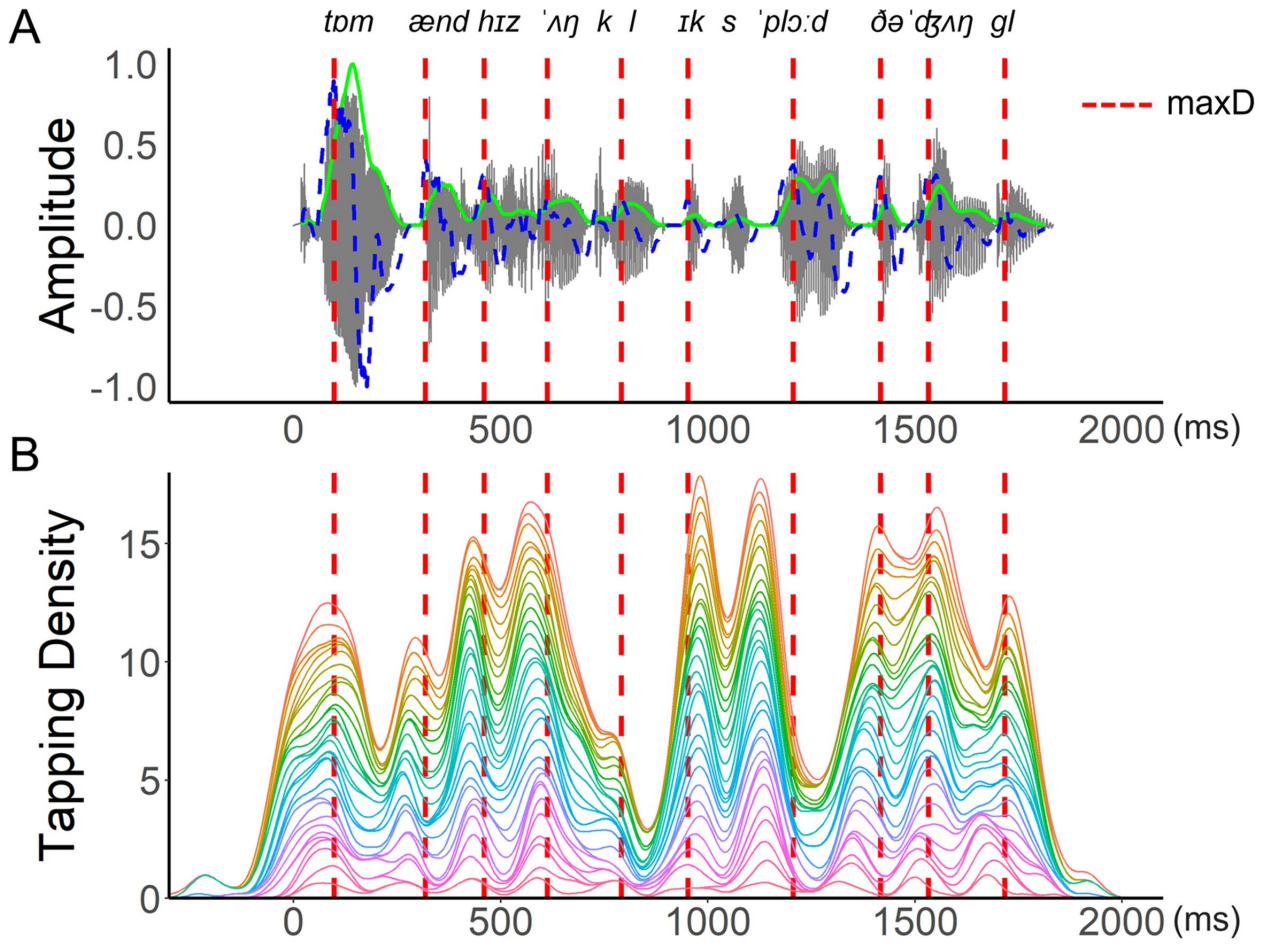
An example of aggregated tapping density. An example of **(A)** the waveform (grey lines), smoothed energy (green lines), and energy difference function (blue dashed lines) of the test sentence “*Tom and his uncle explored the jungle*.” SMS of all participants for this sentence is represented by the aggregated tapping density in **(B)**, with each colored curve showing one participant’s scaled density function (area under the curve = 1). Locations of maxD are indicated by red dashed lines across both panels. Absolute asynchrony is defined as the interval between each tapping peak and its nearest maxD location. Signed asynchrony refers to the same interval duration but is positive for taps that follow a landmark and negative for taps that precede it.

The location of maxD was used as the synchronization landmark for tapping peaks produced nearby. A tapping peak within a ± 120 ms window around a maxD landmark was treated as anchored to that landmark, and asynchronies were calculated (Repp, 2004). Both absolute and signed asynchronies were analysed. Absolute asynchronies reflected the duration of intervals between a tapping peak and a nearby maxD landmark (see Figure 5), and served as a measure of overall synchronization accuracy. Signed asynchronies reflected the exact timing of a tap relative to the landmark set at 0 ms and characterized tapping peaks as either preceding or following a nearby landmark in time (Rathcke and Lin, 2021; Rathcke et al., 2019; Rathcke et al., 2021b). In addition to measuring synchronization accuracy, signed asynchronies reflect anticipatory tendencies during SMS (Aschersleben, 2002; Repp, 2005).

Given the main focus of the current research on the modulations of syllable rise-time and their role in SMS with a selection of naturally spoken sentences, the analyses below are based on an aggregated dataset comprising the median of all participants’ tapping asynchronies calculated relative to the maxD of each syllable. As indicated in Figure 5, individuals vary in their ability to tap precisely in time with an acoustic landmark, though at the group level, there is a general pattern of synchronization indicating if participants tended to tap close to a local landmark or further away from it. The by-item analysis chosen for the present study therefore focused on SMS responses to the tests sentences and reduced the impact of individual variation typically observed in SMS due to interpersonal differences in musical training, spontaneous motor tempo, and other potential factors (Rathcke et al., 2024; Rathcke et al., 2021b). By-participant aggregation is a common practice in the analyses seeking to simplify the data structure and to highlight general trends (e.g., Dixon and Cunningham, 2006; Jesus et al., 2015).

### 2.7 Analyses

All analyses reported below were conducted in R (version 4.5.0). The non-transformed values of absolut e asynchronies showed a left-skewed distribution peaking around 40 ms (*W* = 0.95, *p* < 0.05) while the log-transformed values were normally distributed around 3.7 (*W* = 0.997, *p* = 0.71; cf. Baayen, 2008). In contrast, the distribution of raw signed asynchronies was normal (*W* = 0.997, *p* = 0.71) and did not require further pre-processing. Raw signed asynchronies and log-transformed absolute asynchronies entered linear regressions as dependent variables.

Linear regressions were separately performed for the three dependent variables of interest: (1) syllable rise-time, (2) absolute asynchronies, and (3) signed asynchronies. In the first set of models, rise-slope, nucleus duration, metrical weight, onset complexity, phonological sonority, intensity, and serial order were fit as predictors to the rise-time data calculated for each syllable. In the second and the third set of models, rise-time, rise-slope, nucleus duration, metrical weight, onset complexity, phonological sonority, intensity, and serial order were fit as predictors to the median absolute and signed asynchronies calculated for each syllable, respectively. Sentences were initially modeled as random intercepts in all models. However, there was no variance arising from the sentence factor in any of the models. Thus, a linear regression was performed and is reported in the results section. To reduce the skewness of the distribution of duration, absolute asynchronies and rise-times were logarithmically transformed (cf. Baayen, 2008).

**Figure 6:**
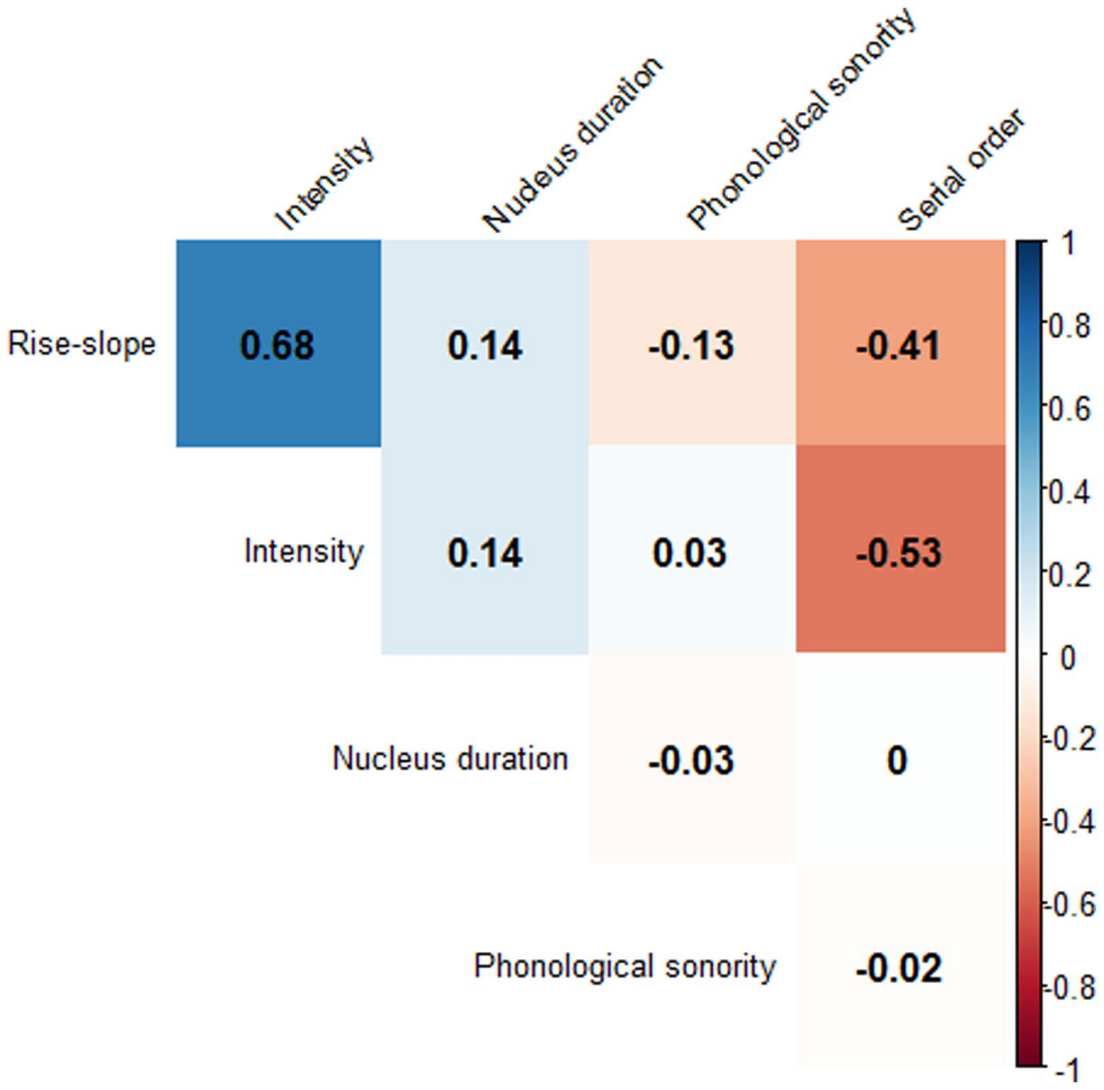
Correlation matrix of all numerical measurements used to predict rise-time variability.

The modeling started with a fully specified model that included all predictors, with the non-significant ones being subsequently removed. The procedure continued until a best-fit model was established. A likelihood-ratio test between the initial full model and the final model was conducted to ensure that the initial full model did not outperform the final best-fit model. No major collinearity issues among the predictors were observed in these data. The variable inflation factors (VIFs) were all smaller than 2 in all models, below the common cut-off value of 3 (Thompson et al., 2017). A correlation matrix is given in Figure 6 to outline correlations between the predictors. Serial order was negatively correlated with intensity and rise-slope; onset complexity was negatively correlated with phonological sonority; and rise-slope was positively correlated with intensity (the alpha rate was adjusted to 0.001 because of multiple tests). All data and the analysis code can be found on OSF (https://osf.io/gdq96/).

## 3 Results

### 3.1 Factors influencing rise-time

**Figure 7:**
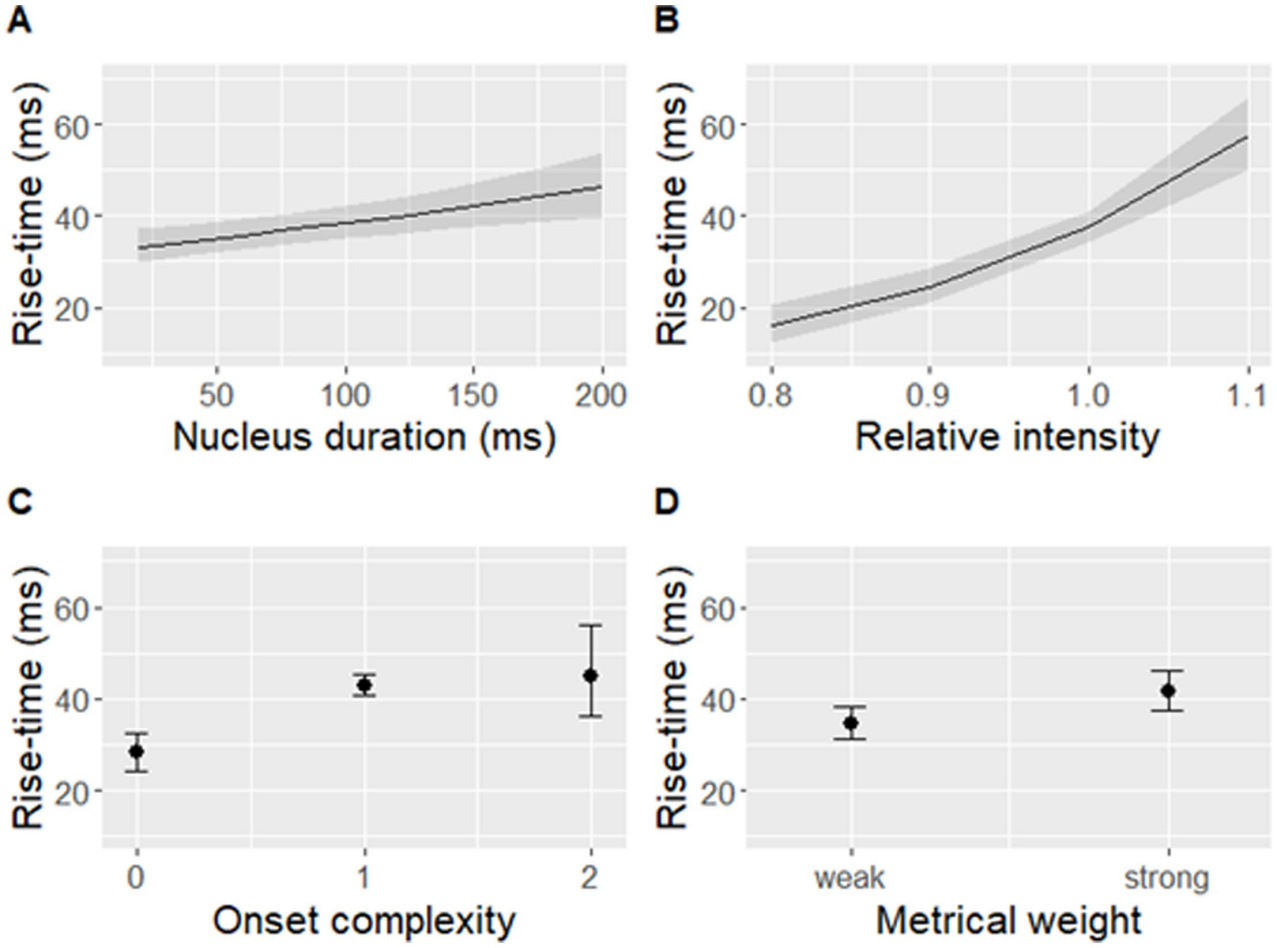
Rise-time predicted by **(A)** nucleus duration, **(B)** relative nucleus intensity, **(C)** onset complexity, and **(D)** metrical weight of a syllable. Shaded bands **(A,B)** and error bars **(C,D)** indicate 95% confidence intervals. Note that rise-time was log-transformed in the model but is displayed on the original scale in this figure.

The best-fit model significantly predicted log-transformed rise-time [*F*(5,446) = 40.23, *p* < 0.001] based on metrical weight, onset complexity, vowel duration, and intensity (Table 1 and Figure 7).

**Table 1:** Results of the best-fitting linear regression model of rise-time.

Factor	*b*	SE	*t*	95% confidence interval		β
Intercept	3.25	0.08	42.59**	3.10	3.40	—
Metrical weight: Strong^a^	0.18	0.06	3.17*	0.07	0.30	0.12
Intensity	0.20	0.03	7.49**	0.15	0.25	0.12
Onset complexity: 1^b^	0.42	0.08	5.36**	0.27	0.58	0.13
Onset complexity: 2^b^	0.47	0.13	3.50**	0.21	0.74	0.17
Nucleus duration	0.09	0.03	3.44**	0.04	0.14	0.06

^a^Reference level is Weak; ^b^Reference level is 0 (i.e., no onset consonants); *p = <0.01; **p = <0.001.

### 3.2 Absolute asynchrony of SMS

**Figure 8:**
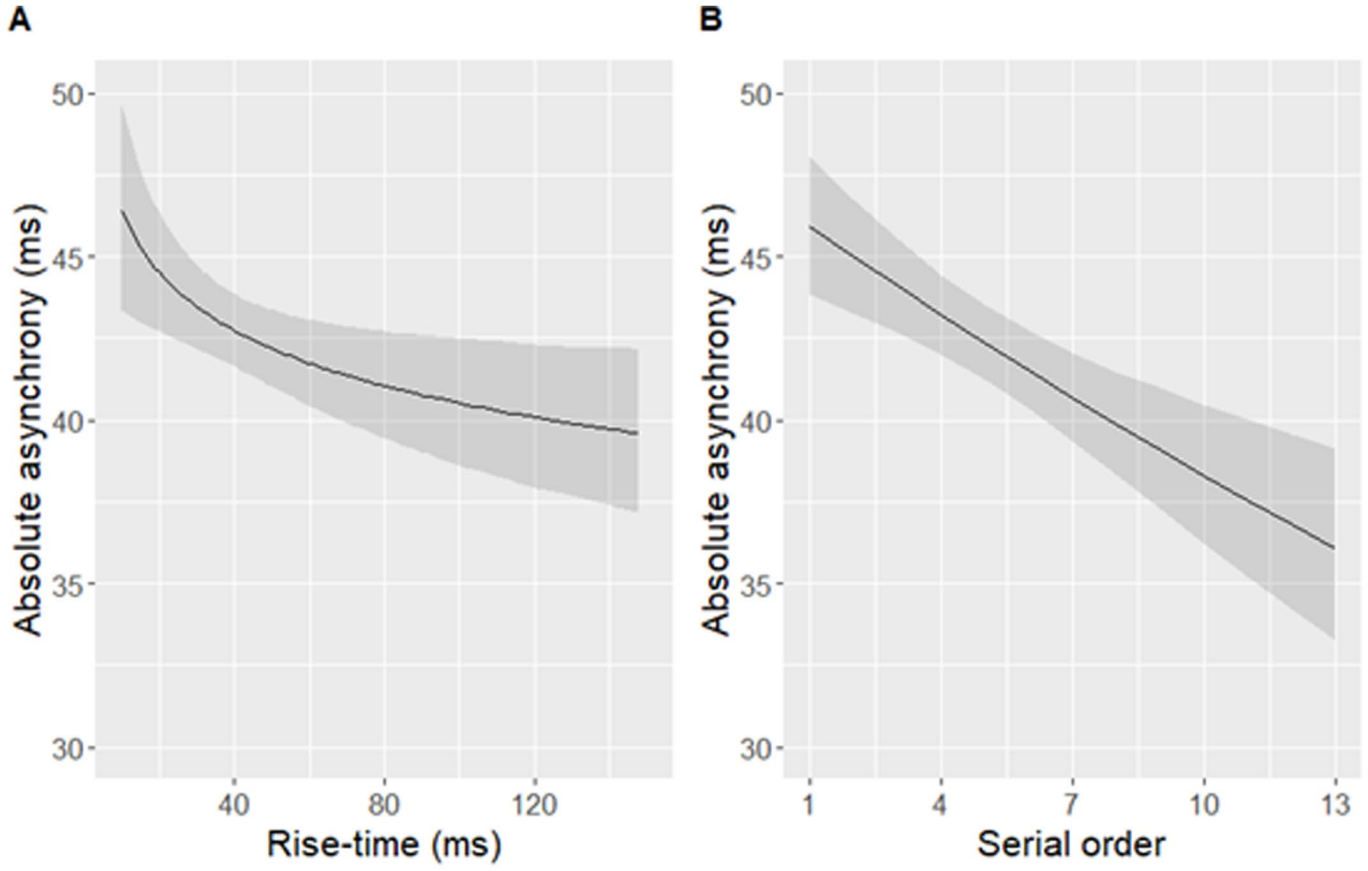
Absolute asynchronies predicted by **(A)** rise-time **(B)** the serial order of a syllable within a sentence. Note that a larger asynchrony indicates that a tapping peak was further away from an adjacent landmark occurring within the predefined window of ±120 ms around maxD. Shaded bands indicate 95% confidence intervals. Rise-time was log-transformed in the model but is displayed on the original scale in the figure.

The best-fit model significantly predicted log-transformed absolute asynchronies in the aggregated dataset [*F*(2,449) = 11.03, *p* < 0.001]. Log-transformed rise-time and the serial order of a syllable in a sentence significantly predicted log-transformed absolute asynchronies of syllables (Table 2 and Figure 8). Participants’ taps were closer to the P-centre approximation (maxD) in syllables with longer rise-times, and occurring later in a sentence. The final, more parsimonious model fit the data as well as the initial, fully specified model [*F*(6,443) = 0.82, *p* = 0.56].

**Table 2:** Results of the best-fitting linear regression model of absolute asynchrony.

Factor	*b*	SE	*t*	95% of confidence interval		*β*
Intercept	4.07	0.09	43.68**	3.89	4.25	—
log(rise-time)	−0.06	0.02	−2.60*	−0.10	−0.01	−0.10
Serial order	−0.02	0.005	−4.16**	−0.03	−0.01	−0.07

*p < 0.01; **p = <0.001.

### 3.3 Signed asynchrony of SMS

**Figure 9:**
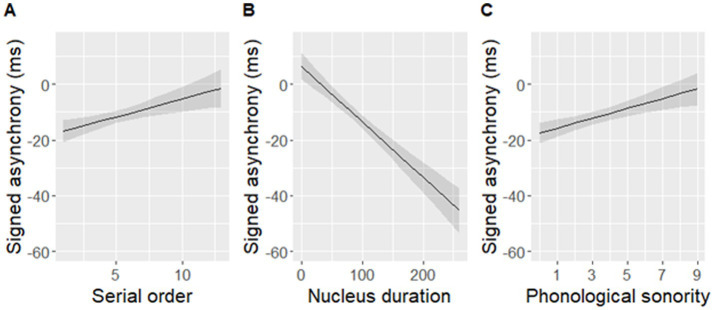
Signed asynchronies predicted by **(A)** serial order, **(B)** nucleus duration, and **(C)** phonological sonority of a syllable within a sentence. Note that a negative signed asynchrony indicates that a tapping peak occurred earlier than an adjacent landmark within the predefined window of ±120 ms around maxD while a positive signed asynchrony indicates that a tapping peak occurred later than the adjacent landmark. Shaded bands indicate 95% confidence intervals.

The best-fit model significantly predicted signed asynchronies measured in the data [*F*(3,448) = 32.63, *p* < 0.001], but it did not include an effect of either rise-time or rise-slope. Instead, nucleus duration, phonological sonority, and serial order predicted signed asynchronies (Table 3 and Figure 9). The final model with the three predictors fit the data as well as the initial model with all seven predictors [*F*(6,442) = 2.07, *p* = 0.06].

**Table 3:** Results of the best-fitting linear regression model of signed asynchrony.

Factor	*b*	SE	*t*	95% of confidence interval		*β*
Intercept	−18.15	2.35	−7.74**	−22.76	−13.54	—
Nucleus duration	−9.74	1.14	−8.56**	−11.98	−7.51	−0.37
Phonological sonority	4.24	1.14	3.73**	2.01	6.48	0.16
Serial order	1.29	0.40	3.18*	0.49	2.08	0.14

*p < 0.01; **p = <0.001.

## 4 Discussion

The present study focused on the rise-time of amplitude envelopes in natural speech. Using a set of 52 English sentences (with a total of 452 syllables in the analysis), we investigated (1) which linguistic and/or acoustic factors may shape the duration of syllable rise-times in connected speech, and (2) how naturally variable rise-times (along with other linguistic and acoustic factors) may influence sensorimotor synchronization with connected speech. Two main findings emerged from this investigation. First, rise-time varied significantly in response to varying degrees of metrical weight, nucleus duration, and intensity, as well as onset cluster complexity in the corresponding syllable. Second, syllable rise-time significantly predicted absolute asynchrony with the P-centre of each syllable (approximated by the location of maxD, Šturm and Volín, 2016) but did not play a role in predicting signed asynchrony.

As hypothesized, the results showed that rise-time of amplitude envelopes was the result of a complex interplay between several acoustic signal features (relative intensity and duration of a syllable nucleus) and linguistic factors (onset complexity and metrical weight of a syllable) that exerted independent influences on the duration of syllable rise-time. The study provided evidence that, in natural speech, rise-time tends to increase with higher number of onset consonants, higher intensity and longer duration of the nucleus, as well as higher metrical weight of the syllable. The effect of high metrical weight on prolonging rise-time is in full alignment with previous research using isolated English words (Leong et al., 2011) while the effect of the consonant type (e.g., sonorant vs. obstruent) that has been previously suggested to influence rise-time in isolated syllables controlled for vowel quality and onset complexity (Goswami et al., 2010) was absent in the present data – possibly outweighed by other factors under investigation that had a more consistent effect. The relative intensity and nucleus duration are themselves hallmarks of the linguistic structure and carry multitudes of linguistic meanings. For example, duration can reflect word boundaries (Turk and Shattuck-Hufnagel, 2000) and, more generally, the structure of the prosodic hierarchy (Aylett and Turk, 2004) while intensity varies with open vs. close vowel quality (Bladon and Lindblom, 1981).

Taken together, these findings indicate that, in natural connected speech, amplitude rise-time does not have a clear one-to-one mapping to a specific linguistic function or a specific feature of the acoustic speech signal and is therefore difficult to interpret meaningfully. In a similar vein, a recent study of lexical stress perception in Italian has shown that both dyslexic and typically developing listeners failed to use experimentally manipulated duration of amplitude rise-time as a reliable acoustic cue to lexical stress while distinguishing word pairs like *pápa* (meaning ‘pope’) vs. *papá* (meaning ‘dad’; Rossi and Rathcke, 2025). We therefore suggest that studies of clinical and typically developing populations ought to be cautious in drawing conclusions about the nature of perceptual processing and potential deficits when relying on data collected using holistic acoustic measures such as rise-time, given the one-to-many relationship between amplitude rise-time and its potential language-specific functions.

As far as the role of rise-time in sensorimotor synchronization is concerned, the present results showed that a group of 31 native English participants were less precisely synchronized to the P-centre (measured as maxD; Šturm and Volín, 2016) in syllables with shorter rise-times than in syllables with longer rise-times. Surprisingly, the direction of this effect is at odds with the results reported in a previous finger-tapping study showing the opposite pattern (Rathcke et al., 2021b), and also somewhat at odds with the research on non-speech (e.g., tonal) stimuli, showing that shorter rise-time and steeper rise-slope reduced the amount of negative mean asynchrony (i.e., tapping ahead of the stimulus; London et al., 2025; Vos et al., 1995). Moreover, rise-time did not show an effect on signed asynchrony in the present study, even though it played a significant role in a smaller set of previously investigated sentences (Rathcke et al., 2021b).

Several reasons may account for the diverging directions of the rise-time effect. Since the present study focused on the understanding of the key properties of syllables with variable rise-times, the measurement of SMS involved aggregated data and used the median of asynchronies to represent average synchronization performance with each syllable. In contrast, our previous work focused on the understanding of the key properties of SMS and used one measurement per participant and syllable (Rathcke et al., 2021b). An item-based analysis of aggregated data may have oversimplified the relationship between syllabic rise-time and tapping asynchronies, given that individual differences were not estimated. Nevertheless, aggregating tapping responses across participants is a widely used approach in SMS research, particularly in studies involving tempo perturbations or non-isochronous stimuli, where the aim is to uncover consistent group-level patterns rather than individual-level variability (e.g., Darabi and Svensson, 2021; López and Laje, 2019; Repp and Moseley, 2012). By-participant aggregation also represents a common analytical strategy to simplify data structure and highlight general trends (e.g., Dixon and Cunningham, 2006; Jesus et al., 2015). In the context of the present study, we were primarily interested in the general patterns of rhythmic motor alignment to the amplitude modulation of natural speech and focused on item-level features and group-level behavior, rather than exploring inter-individual variation.

More importantly, the present study was based on 52 English sentences and included 452 syllables in the analyses while our previous work piloted the sensorimotor synchronization paradigm with only 6 sentences and 42 syllables in total (Rathcke et al., 2021b). A larger set of materials was expected to cover a wider range and variability of rise-time, thus providing a more representative account of the role of rise-time in rhythm perception as captured by SMS. In contrast to the experiment by Vos et al. (1995), which manipulated duration and intensity of tonal stimuli and the changing properties of the resulting rise-times and tested synchronization with tones separated by short acoustic silences, the present study involved synchronization with complex natural speech stimuli. Here, intensity and duration may often be interrelated (Leong et al., 2011). For example, the presence of increased metrical weight often (but not always; e.g. Campbell and Beckman, 1997) goes hand in hand with an increase in vowel duration (van Santen, 1992) and intensity (Fry, 1955), though their contribution to the properties of rise-time has not been systematically investigated to date.

To conclude, the present study provided evidence that the duration of syllable rise-time in natural speech was influenced by a number of acoustic and linguistic factors shaping the amplitude envelope of naturally spoken sentences. In small datasets such as the aggregated tapping data used here (and many clinical datasets), the syllable rise-time may emerge as a stronger predictor of sensorimotor synchronization (esp. absolute asynchrony) as compared to more linguistically interpretable predictors related to the prosodic and segmental structure of speech, such as metrical weight or onset complexity. And yet, it is the role of the linguistic structure that is of utmost importance to the study of rhythm perception in clinical and healthy populations (Beckman, 1992; Rathcke and Lin, 2021; Rathcke and Smith, 2015), reducing the universal usefulness of holistic acoustic signatures such as rise-time in the context of rhythm research.

## Data Availability

The datasets presented in this study can be found in online repositories. The names of the repository/repositories and accession number(s) can be found in the article/supplementary material.
